# Sodium-Glucose Co-transporter 2 Inhibitors/Gliflozins: A New Miracle Therapy for Heart Failure Patients Irrespective of Diabetes Status?

**DOI:** 10.7759/cureus.31777

**Published:** 2022-11-22

**Authors:** Paghunda Ehsan, Rawia N Aburumman, Shivani Ishwarya Muthanna, Swathi Radhakrishnan Menon, Vruti Vithani, Bansi Sutariya, Diana M Montenegro, Michael Chukwu, Sai Sri Penumetcha

**Affiliations:** 1 Internal Medicine, Hayatabad Medical Complex, Peshawar, PAK; 2 Internal Medicine, Lady Reading Hospital, Peshawar, PAK; 3 Research, California Institute of Behavioral Neurosciences & Psychology, Fairfield, USA; 4 Internal Medicine, Mutah University, Amman, JOR; 5 Internal Medicine, Vydehi Institute of Medical Sciences and Research Center, Bangalore, IND; 6 Pediatrics, Smt. Kashibai Navale Medical College and General Hospital, Pune, IND; 7 Internal Medicine, California Institute of Behavioral Neurosciences & Psychology, Fairfield, USA; 8 General Medicine, Government Medical College, Surat, IND; 9 General Surgery, California Institute of Behavioral Neurosciences & Psychology, Fairfield, USA; 10 General Surgery, Pilgrim Hospital, Boston, GBR; 11 General Medicine, Chalmeda Anand Rao Institute of Medical Sciences, Karimnagar, India, IND

**Keywords:** global health education, acute decompensated heart failure, heart failure with reduced ejection fraction, heart failure with preserved ejection fraction (hfpef), hypertrophic obstructive cardiomyopathy (hocm), st-elevation myocardial infarction (stemi), heart failure, dapagliflozin, empagliflozin, sodium-glucose co-transporter-2 inhibitors

## Abstract

Despite the existence of effective medicines, heart failure continues to be the largest cause of illness and death worldwide. As a prospective family of drugs with potential cardiovascular advantages in non-diabetic patients, sodium-glucose co-transporter 2 inhibitors (SGLT2-I) have recently come to the forefront. In this comprehensive study, we assessed the favorable cardiovascular outcomes of SGLT2-I in three sizable, randomized trials with both diabetic and non-diabetic populations. The results from these studies revealed a substantial reduction in heart failure hospitalizations and cardiovascular and all-cause deaths. To further support our assertion that SGLT2-I has the potential to be a novel addition to the standard treatment plan for heart failure, we also tried to assemble several post hoc and prespecified studies of the Dapagliflozin and Prevention of Adverse Outcomes in Heart Failure (DAPA-HF) study. The details of two clinical investigations that supported their use in acute decompensated heart failure were also examined, along with the most plausible mechanism of action generating their cardioprotective effects. Additionally, positive cardiovascular advantages were addressed in chronic heart failure with both preserved and reduced ejection fractions. The role of SGLT2-I in ST-elevation myocardial infarction (STEMI) and hypertrophic cardiomyopathy (HOCM) patients is currently being studied, and this research has the potential to be revolutionary. The purpose of this systematic review is to compile all information that supports the use of this life-saving drug in patients who do not have diabetes so that cardiac care can be improved globally.

## Introduction and background

The need for innovations in heart failure (HF) therapy is critical due to the enormous morbidity and death linked to the condition. Worldwide, the number of instances of cardiovascular disease nearly quadrupled from 271 million to 523 million between 1990 and 2019. Cardiovascular mortality increased as well, from 12.1 million in 1990 to 18.6 million in 2019 [1]. There are certain groups of medications used conventionally for the prevention and management of heart failure, including angiotensin-converting enzyme inhibitors, angiotensin II receptor blockers, diuretics, neprilysin inhibitors, nitrates, and beta blockers. However, there is still room for new medications to fall into this category.

First identified as a novel anti-diabetic medication, sodium-glucose cotransporter-2 inhibitors (SGLT2-I) were introduced into the market after multiple clinical trials proved their magical role in appropriate diabetic control. Canagliflozin (CANA) was approved by the Food and Drug Administration (FDA) in March 2013 as the first SGLT2 inhibitor, followed by dapagliflozin (DAPA) and, empagliflozin (EMPA) in 2014 [2]. However, recent studies have shown that SGLT2-I are effective in improving negative cardiovascular (CV) and renal outcomes in diabetic patients [3-6]. The mechanisms through which SGLT2-I exhibit cardioprotective effects are postulated as activation of anti-inflammatory and oxidative stress pathways, increased ketone body production and decreased advanced glycation end products [7,8], and reduction in intracellular fluid volume owing to osmotic diuresis [9].

In recent years, studies have been done to see if SGLT2-I treatment has any favorable cardiovascular effects, even in individuals with HF who are not diabetic. Beginning with two large, randomized trials, the Dapagliflozin and Prevention of Adverse Outcomes in Heart Failure (DAPA-HF) and the Empagliflozin Outcome Trial in Patients With Chronic Heart Failure With Reduced Ejection Fraction (HFrEF) (the EMPEROR-Reduced trial), which included both diabetic and non-diabetic population, showed a significant reduction in the incidence of heart failure events [10-12]. Additionally, Empagliflozin, Health Status, and Quality of Life in Patients With Heart Failure and Preserved Ejection Fraction (the EMPEROR-Preserved trial), which had non-diabetic heart failure patients with preserved ejection fraction (HFpEF), and the EMPULSE trial (the SGLT2 inhibitor empagliflozin in patients hospitalized for acute heart failure: a multinational randomized trial), which involved non-diabetic patients with acute heart failure, both showed encouraging results for symptom relief and health-related quality of life [13,14].

This systematic review's goal is to compile all the information that supports SGLT2-I's favorable therapeutic effects on non-diabetic heart failure patients so that it can be utilized as a standard for future studies and enhance the quality of care for cardiac patients globally.

## Review

Methods

The inclusion and exclusion criteria, the search method used, the bias calculation in each research, and the selection of studies are discussed in this section.

Inclusion and Exclusion Criteria

Table 1 summarizes the inclusion criteria for this review paper. Systematic reviews, meta-analyses, books and documents, grey literature, non-human studies, studies conducted before 2017, and studies conducted in languages other than English that did not fulfill all the inclusion criteria listed in Table 1 were excluded.

**Table 1:** Inclusion Criteria SGLT2-I = Sodium-glucose Cotransporter-2 (SGLT2) Inhibitors

Type of studies	Subject inclusion criteria	Intervention of interest
Randomized control trials and clinical trials done within the last five years.	Middle-aged males 45 to 80 years and above who were heart failure patients (acute heart failure or chronic heart failure with reduced or preserved ejection fraction) whether they had concurrent diabetes or not.	SGLT2-I used as a treatment regimen for heart failure patients whether they were diabetic or not.

Search Strategy

Two research databases including PubMed and Cochrane library were extensively used to find literature on the subject. All databases were last accessed on June 28, 2022. As shown in Table 2, the search was conducted using both common keywords and MeSH (Medical Subject Heading) keywords depending on the type of database used.

**Table 2:** Search Strategy Utilizing Several Databases PubMed: Public/Publisher MEDLINE

Database	Keywords	Filter criteria	Search results
PubMed	Sodium-glucose co-transporter 2 inhibitors OR Empagliflozin OR Dapagliflozin OR Canagliflozin OR Ertugliflozin OR ( "Sodium-Glucose Transporter 2 Inhibitors/pharmacology"[Majr] OR "Sodium-Glucose Transporter 2 Inhibitors/standards"[Majr] OR "Sodium-Glucose Transporter 2 Inhibitors/therapeutic use"[Majr] ) AND Heart failure OR Cardiac failure OR ( "Heart Failure/drug therapy"[Majr] OR "Heart Failure/therapy"[Majr] )	Studies included: Randomized control trials and clinical trials Publication years: 2017-2022	2466
Cochrane Library	Sodium-glucose co-transporter 2 inhibitors AND heart failure	Studies included: Clinical trials Years: 2017-2022	150

To eliminate duplicate references, the ones retrieved from databases were alphabetically sorted using Microsoft Excel 2022 (Microsoft Corporation, Redmond, WA). The studies were further scrutinized using the titles and abstracts to filter out any that did not fit the inclusion criteria. Based on the titles and abstracts, the records were examined, removing studies that were not relevant. Retrieval of the full-text articles came next, after reviewing.

Quality Appraisal of Individual Studies

All publications that satisfied the inclusion requirements underwent risk of bias assessment using instruments designed specifically for the original study type. As shown in Table 3, the Cochrane Collaboration Risk of Bias Tool (CCRBT) was used for randomized clinical trials (RCTs) and Newcastle Ottawa Scale (NCOS) for the non-randomized clinical trials (NRCTs) [15,16].

**Table 3:** Risk of Bias Assessment RCT: Randomized Clinical Trial; CCRBT: Cochrane Collaboration Risk of Bias Tool; NCOS: Newcastle Ottawa Scale; NRCT: Non-randomized Clinical Trial

Quality assessment tool	Study type	Total score	Accepted score >70%	Studies accepted
CCRBT [15]	RCTs	7	5	15
NCOS [16]	NRCTs	8	6	01

Selection of Individual Studies

Two authors (Paghunda Ehsan and Rawia N. Aburumman), who participated in the study selection process, performed an initial screening of all papers that satisfied the inclusion criteria by reading their titles and abstracts. Studies in English and those which included non-diabetic heart failure patients taking SGLT2-I for heart failure treatment were accepted. In the second stage of screening, the papers that were determined to be pertinent to the research issue were further examined. RCTs and NRCTs that met the requirements for inclusion and demonstrated minimal bias risk on the quality assessment tools were then subjected to full-text review.

**Figure 1:**
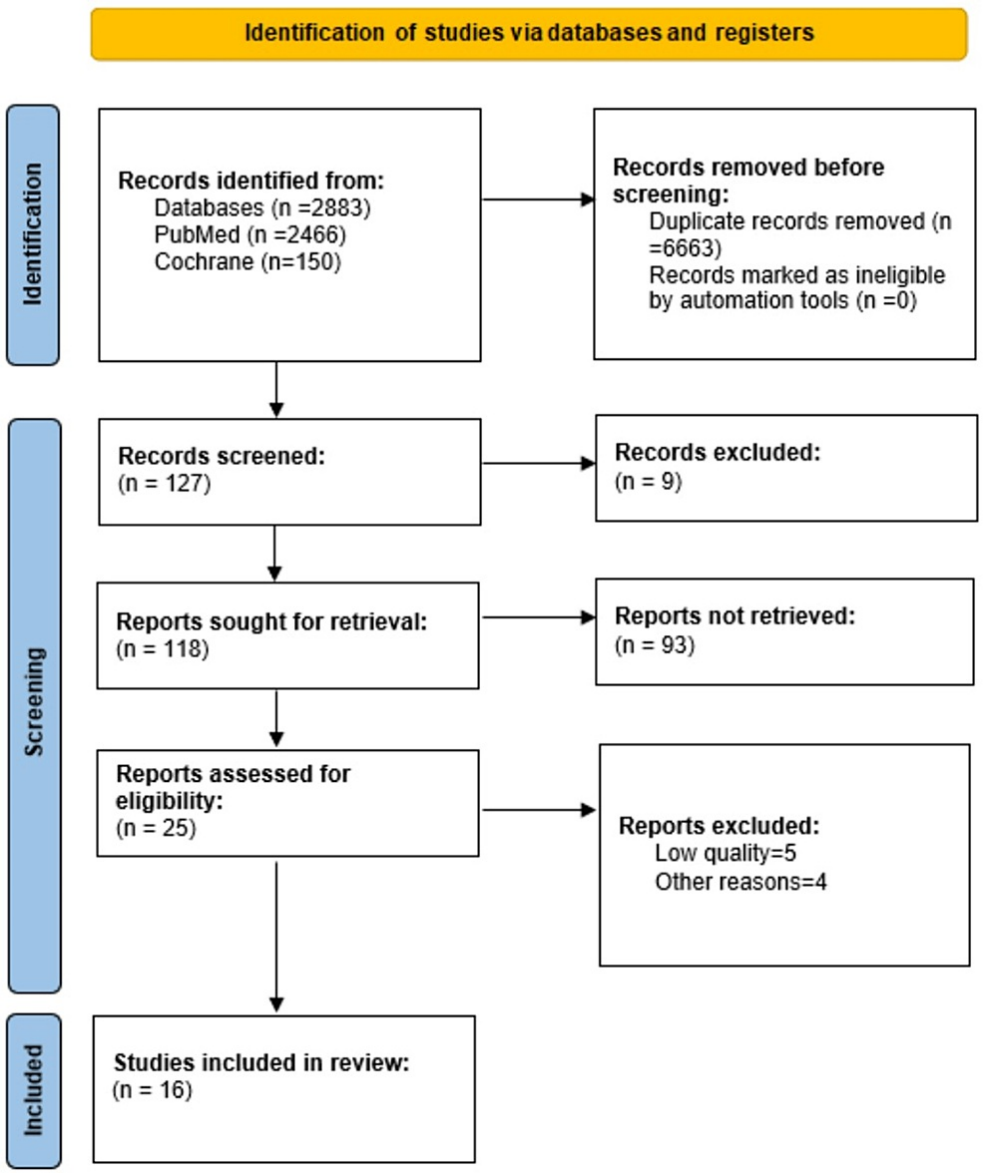
Comprehensive PRISMA Flow Diagram PRISMA: Preferred Reporting Items for Systematic Reviews and Meta-Analyses

This systematic review was conducted based on the Preferred Reporting Items for Systematic Reviews and Meta-Analyses (PRISMA) 2020 guidelines and the search strategy is outlined in Figure 1 [17]. We followed PRISMA 2020 recommendations [18].

Results

A total of 2833 studies were obtained after searching using two databases, namely PubMed and Cochrane Library, using the search strategy described. 6663 duplicates were removed using Microsoft Excel 2022 version. Of the 2833 studies, 127 articles were screened after duplication removal and removal of records marked ineligible owing to exclusion criteria. Nine studies were excluded as their topics were irrelevant. The abstracts of the remaining 118 articles were thoroughly read by the author to select the studies which fit the research question better. Out of 118 articles, only 16 articles managed to match the inclusion criteria of the study and were included in this systematic review, out of which characteristics of 14 articles are outlined in Table 4 below.

**Table 4:** Brief Overview of Randomized and Non-Randomized Clinical Trials Included in this Systematic Review DAPA: Dapagliflozin; IF: Interstitial Fluid; SGLT2-I: Sodium-glucose co-transporter 2 inhibitors; DAPA-HF: Dapagliflozin and Prevention of Adverse Outcomes in Heart Failure; HF: Heart Failure; CV: Cardiovascular; RCT: Randomized Clinical Trial; HFrEF: Heart Failure with Reduced Ejection Fraction, EMPA-TROPISM: Are the "cardiac benefits" of Empagliflozin independent of its hypoglycemic activity; LV: Left Ventricle ; QoL: Quality of Life; EMPEROR- PRESERVED: Empagliflozin Outcome Trial in Patients with Chronic Heart Failure with Preserved Ejection Fraction; EMPA: Empagliflozin; KCCQ: Kansas City Cardiomyopathy Questionnaire; EMPULSE; Empagliflozin in Patients Hospitalized for Acute Heart Failure; EMPEROR-REDUCED: Empagliflozin Outcome Trial in Patients with Chronic Heart Failure with Reduced Ejection Fraction; PCWP: Pulmonary Capillary Wedge Pressure; DEFINE-HF: Dapagliflozin Effects on Biomarkers, Symptoms, and Functional Status in Patients with HF with Reduced Ejection Fraction; EF: Ejection Fraction; EMPA-AHF: Empagliflozin in Acute Heart Failure

Reference/Article	Article type	Year	SGLT2-I used	Total sample size	Non-diabetic sample	Objective	Conclusion
Hallow et al. [9]	Clinical Trial	2017	DAPA	42	42	Osmotic diuresis caused by SGLT2-I causes a greater electrolyte-free water clearance and, ultimately, a greater fluid clearance from the interstitial fluid (IF) space than from the bloodstream.	SGLT2 inhibitors offered superior control of congestion without affecting arterial filling and perfusion.
McMurray et al. [10]	RCT (DAPA-HF)	2019	DAPA	4744	2605	To evaluate the effects of SGLT2-I in pts with HFrEF independent of diabetes status.	DAPA recipients had a decreased risk of worsening heart failure or dying from cardiovascular causes than those who got a placebo.
Petrie et al. [11]	RCT (DAPA-HF) Exploratory analysis	2020	DAPA	4744	2605	Whether or not a patient has diabetes, it is important to determine if DAPA has an impact on their cardiovascular outcomes.	Reduction in worsening of HF and CV death was noted in non-diabetics just like diabetics, in addition to the recommended therapy.
Santos-Gallego et al. [12]	RCT (EMPA-TROPISM)	2021	EMPA	84	84	To evaluate how empagliflozin affects non-diabetic HFrEF patients' left ventricular (LV) performance and volumes, functional ability, and quality of life (QoL).	The EMPA-TROPISM trial showed EMPA's superiority over placebo in aspects of LV volumes, mass, and systolic function, functional capacity, and quality of life in non-diabetic HFrEF patients.
Butler et al. [13]	RCT (EMPEROR-PRESERVED)	2022	EMPA	5942	3031	To assess if clinical benefit seen with EMPA changes based on baseline health status, and to assess the impact of EMPA on health-related quality of life in HF with preserved ejection fraction pts.	EMPA decreased the risk for serious heart failure outcomes in patients with HF with preserved ejection fraction across the range of KCCQ scores along with early and long-lasting improvement in health-related quality of life.
Voors et al. [14]	RCT (EMPULSE)	2022	EMPA	530	290	To evaluate if empagliflozin improves clinical outcomes when initiated in patients who are hospitalized for acute heart failure regardless of diabetes status.	By emphasizing on patients hospitalized for acute heart failure, the results of EMPULSE enhance and augment those of EMPEROR-Reduced and EMPEROR-Preserved trials.
Koenigsberger et al. [19]	RCT (DAPA-HF)	2021	DAPA	4744	2605	To demonstrate the findings of DAPA-HF Trial in non-diabetic HF patients.	This study supported the use of dapagliflozin in patients with HF rather than just those with diabetes, who were the target audience for the medication's initial marketing.
Omar et al. [20]	RCT	2020	EMPA	70	54	To study the effects of empagliflozin on central hemodynamics in patients with HF and HFrEF.	In patients with stable HFrEF, empagliflozin for 12 weeks reduced PCWP compared with placebo.
Kolwelter et al. [21]	RCT	2021	EMPA	74	74	To examine empagliflozin's impact on vascular function in HF patients.	EMPA improved vascular function by reducing left ventricular afterload.
Martinez et al. [22]	RCT (DAPA-HF)	2020	DAPA	4744	2605	To evaluate dapagliflozin according to age, given potential concerns about the efficacy and safety of therapies in the elderly.	In the wide range of ages examined by DAPA-HF, dapagliflozin relieved symptoms and decreased the risk of mortality and worsening heart failure.
Berg et al. [23]	RCT (DAPA-HF) Secondary analysis	2021	DAPA	4744	2605	The timing and size of the therapeutic improvement from dapagliflozin as a function of the time since a previous HF hospitalization.	DAPA reduced the relative and absolute risk of cardiovascular mortality or worsening HF in patients with more recent HF hospitalizations.
Butt et al. [24]	RCT (DAPA-HF) Prespecified subgroup analysis	2021	DAPA	4744	2605	To compare DAPA efficacy in male and female HF patients.	Regardless of sex, DAPA equally enhanced the primary outcome and health-related quality of life in HF patients.
Nassif et al. [25]	RCT (DEFINE-HF)	2019	DAPA	236	70	To establish the effect of DAPA in established HF with reduced EF in patients with or without diabetes.	The advantages of DAPA on clinically meaningful HF measures in patients with HFrEF extend to patients without diabetes as well.
Damman et al. [26]	RCT (EMPA-AHF)	2020	EMPA	79	11	To study the effects of EMPA in acute decompensated HF patients.	EMPA reduced a combined endpoint of in-hospital worsening HF, rehospitalization for HF or death at 60 days compared with placebo.

Discussion

This comprehensive review firmly supports the claim that, regardless of diabetes status, treatment with SGLT2-I lowers the risk of CV death or a composite of deteriorating heart failure (hospitalization or urgent visit leading to intravenous therapy for heart failure). In this section, we will address the most likely mechanism of action and the most recent data on the positive cardiovascular benefits of SGLT2-I in nondiabetic patients. The variations in cardiovascular outcomes across trials, their flaws, and suggested next steps will also be discussed. In the end, it will be clear what must be done more in this area to enhance the cardiovascular outcome for individuals with heart failure.

SGLT2-I Cardioprotective Mechanism of Action

**Figure 2:**
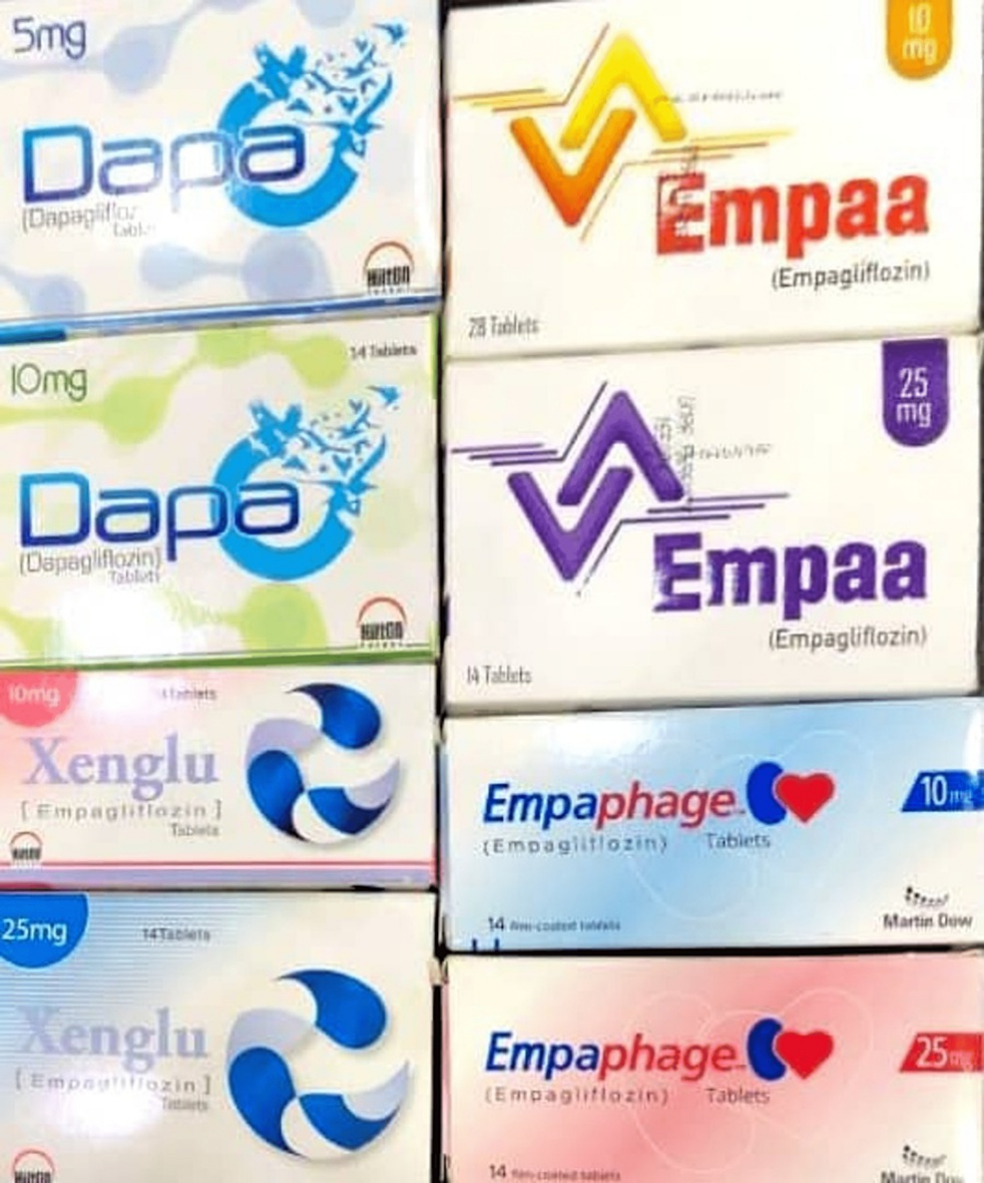
Pakistani Local Brands having Different SGLT2-I Formulations SGLT2-I: Sodium-Glucose Co-transporter 2 Inhibitors

While the precise mechanism of SGLT2-I is unknown, multiple studies have validated a myriad of hypotheses. The advantages of SGLT2-I in the treatment of HF are mostly due to the stimulation of natriuresis and osmotic diuresis which these drugs induce, which improves cardiac output and reduces afterload and preload [6,9,19,27-29]. According to some research, SGLT2-I hypothetically alters some inflammatory pathways, which could reduce or even potentially reverse cardiac fibrosis, hypertrophic cardiomyopathy, and atherosclerosis [27-29]. It has also been speculated that SGLT2-I alters myocardial fuel consumption, contributing to an increase in fatty acid oxidation and a switch to a rise in ketone body consumption [30]. As per 'are the "cardiac benefits" of empagliflozin (EMPA) independent of its hypoglycemic activity?' (EMPA-TROPISM) trial [31], EMPA in non-diabetic chronic HFrEF patients significantly enhances left ventricular (LV) volumes, LV mass, LV systolic function, functional status, and quality of life [12]. Figure 2 shows an original image taken by the author of the common SGLT2-I found in Pakistani pharmacies.

From March 6, 2018, to September 10, 2019, Omar et al. enrolled 70 patients with stable euvolemic HFrEF (54 non-diabetic patients) to receive either 10 mg of EMPA or a matching placebo once daily based on a standard heart failure treatment regimen for 12 weeks to evaluate the hemodynamic effect of SGLT2-I using right heart catheterization (RHC) at rest and during exercise. The ratio of pulmonary capillary wedge pressure (PCWP) to cardiac index (CI) at maximum exertion after 12 weeks was the main result. In contrast to the placebo, the PCWP/CI ratio did not change during exercise or at rest, but PCWP was reduced across the board in all hemodynamic parameters, lowering LV filling pressures [20].

In 2021, Kolwelter et al. randomized 2:1 participants with HF New York Heart Association (NYHA) II-III and an ejection fraction of 49 percent or less to receive EMPA 10 mg once daily or a placebo for three months to determine the impact of EMPA on vascular function. Vascular metrics such as central systolic blood pressure (cSBP), central pulse pressure (cPP), forward pressure height (FPH), and reflected pressure pulse height (RPH) under basal conditions decreased in the EMPA therapy group (n = 45) after one and three months. The decreased afterload of the left ventricle caused by greater arterial and aortic compliance perhaps led to a better prognosis seen in HF patients taking SGLT2 inhibition [21].

Evidence Presently Available, Issues, as well as Future Suggestions for SGLT2-I's Cardiovascular Benefits

The impact of SGLT2-I in chronic heart failure patients in a handful of trials will be reviewed in the upcoming paragraphs.

DAPA-HF Trial

The Dapagliflozin and Prevention of Adverse Outcomes in Heart Failure (DAPA-HF) trial, which involved 4,744 patients, 2,605 of whom did not have diabetes, was the first notable groundbreaking RCT of SGLT2-I to include both diabetic and non-diabetic patients [10]. The trial's participants had HF with low ejection fraction and were monitored for a median of 18.2 months; the major outcomes for those with and without diabetes showed comparable hazard ratios (HRs) (HR of the primary outcome, 0.75 and 0.73, respectively). As per this study, dapagliflozin (DAPA) was equally as effective in treating 55% of people without type 2 diabetes as it was in treating diabetics. Numerous exploratory post-hoc studies and pre-specified subgroup analyses carried out on the DAPA-HF trial have also shown the foregoing cardiac benefits of SGLT2-I, which will now be explored.

A 2020 exploratory analysis of the DAPA-HF trial by Petrie et al. showed a substantial reduction on in the primary composite outcomes of worsening HF or cardiovascular death in patients without diabetes (HR: 0.73 in non-diabetics, 0.74 in people with glycated hemoglobin of at least 5.7 percent, and 0.67 in people with glycated hemoglobin of less than 5.7 percent) [11]. Martinez et al. found that DAPA's benefits were consistent for patients of all ages, even those above 75. Adverse events also showed no variation. Thus, patients with heart failure and a low ejection fraction should not be deterred from receiving DAPA therapy due to their advanced age [22].

The risk of cardiovascular mortality or worsening heart failure rapidly decreased, with particularly high absolute and relative risk reductions in those with more recent heart failure hospitalizations, according to Berg et al. in a secondary analysis of the DAPA-HF study in 2021 [23].

Owing to sex-related variations in pharmacokinetics and pharmacodynamics, women may react to certain HF therapies differently than the male population [24,32,33]. In a prespecified analysis of DAPA-HF in 2021 performed by Butt et al., DAPA reduced the risk of worsening HF to a similar extent in both men and women (HR: 0.73 [95% confidence interval (CI), 0.63-0.85] and 0.79 [95% CI, 0.59-1.06]), and improved symptoms, physical function, and health-related quality of life for the Kansas City Cardiomyopathy Questionnaire clinical summary score and overall summary score similarly in men and women with HF and reduced ejection fraction. In addition, DAPA was safe and well-tolerated irrespective of sex [24].

EMPEROR-Preserved Trial

Improved health status and quality of life as judged by the Kansas City Cardiomyopathy Questionnaire, across all areas and at all measured periods (12, 32, and 52 weeks), were shown by Butler et al. to extend to HFpEF in the "EMPEROR-Preserved study" in 2021 [13].

EMPEROR-Reduced Trial

In 2019, the results of DAPA-HF were mirrored by the "Empagliflozin Outcome Trial in Patients with Chronic Heart Failure and Reduced Ejection Fraction (EMPEROR-Reduced)", with EMPA's effects being similar in diabetics and non-diabetics (HR: 0.72 and 0.78, respectively) [34].

DEFINE-HF Trial

In the clinical trial entitled "Dapagliflozin Effects on Biomarkers, Symptoms, and Functional Status in Patients with HF with Reduced Ejection Fraction (DEFINE-HF)", Nassif et al. included 236 adult ambulatory patients with or without type 2 diabetes mellitus. Compared to the placebo, a significantly higher percentage of patients receiving dapagliflozin for 12 weeks showed clinically significant changes in their functional status, quality of life, or natriuretic peptides [25]. The impact of SGLT2-I in acute heart failure patients in a few trials will be discussed in the following paragraphs.

EMPULSE Trial

In 2022, Voors et al. in the "EMPA in patients hospitalized for acute heart failure (EMPULSE) trial" randomly assigned 524 non-diabetic patients with a primary diagnosis of acute or decompensated chronic heart failure, regardless of LV ejection, to receive either 10mg EMPA or placebo when they were clinically stable (median time from hospital admission to randomization three days), and they were treated for up to 90 days. The main goal was met by EMPA curing more patients than placebo (stratified win ratio, 1.36; 95 percent confidence interval, 1.09-1.68; P- value =0.0054). A five-point or greater difference in variation from baseline in the Kansas City Cardiomyopathy Questionnaire Total Symptom Score at 90 days, or death from any cause, the number of heart failure events, and the time since the first heart failure event, as a hierarchical composite, were deemed clinical benefits [14].

EMPA RESPONSE-AHF Trial

Trials were conducted to determine SGLT2-I's positive effects on individuals with acute heart failure as well, considering growing evidence of its benefits for people with chronic heart failure. In a multicenter pilot study, Damman et al. randomly assigned 80 patients with acute HF with and without diabetes (EMPA RESPONSE-AHF) to receive either EMPA 10 mg/day or a placebo for 30 days. Results revealed that EMPA was safe, increased urine output, and decreased a composite endpoint of worsening HF, rehospitalization for HF, or mortality at 60 days, but it did not affect change in visual analog scale (VAS) dyspnoea, diuretic response, N-terminal {NT}-prohormone brain natriuretic peptide (NT-proBNP), or length of hospital stay [26].

Recent Guidelines

In the past years, numerous guidelines have been added that recommended the use of SGLT2-I in non-diabetic heart failure patients. For instance, In September 2020, UpToDate added SGLT2 inhibitors as an option for patients with continued symptoms of HF despite the use of appropriate primary agents, whether they have type 2 diabetes or not [28]. SGLT2-I was highlighted as a useful supplement in patients with HF who are already receiving beta-blockers, angiotensin receptor, and neprilysin inhibitors, angiotensin-converting enzyme inhibitors, or angiotensin receptor blockers, as well as an aldosterone receptor antagonist, regardless of the presence of diabetes mellitus, in the 2021 update to the "2017 American College of Cardiology (ACC) Expert Consensus Decision Pathway for Optimization of Heart Failure Treatment" [35]. 

*Ongoing Trials*

Currently, new clinical trials are being conducted worldwide to check the efficacy of SGLT2-I in numerous cardiovascular issues like ST-segment elevation myocardial infarction (STEMI), atrial fibrillation, and hypertrophic obstructive cardiomyopathy (HOCM). An interventional clinical trial, namely "Impact of Dapagliflozin on Cardiac Function Following Anterior Myocardial Infarction in Non-Diabetic Patients (DACAMI)" has recently been conducted by Omar Younis at National Heart Institute, Egypt, and completed on June 30, 2022, checking its role in anterior wall ST-elevation myocardial infarction (MI) [36].

Similarly, there is a new clinical trial "Empagliflozin in Hypertrophic Cardiomyopathy (EMPA-REPAIR)", started on June 1, 2022, in Warsaw, Poland, for EMPA administration at the recommended dose of 10 mg per day for 12 months to patients with hypertrophic cardiomyopathy as the suggested intervention. Diabetic patients will not be included in the trial. A total of 250 patients will be randomized in a double-blind method to either EMPA or a placebo. The study's main outcome measure will be the variation in maximum oxygen uptake (VO2 max), as determined by a cardiopulmonary exercise test. Before and after receiving EMPA or a placebo, VO2max, an objective measure of physical performance, will be measured. Secondary outcomes include modifications to the maximal LV wall thickness, LV mass, measures of diastolic dysfunction, degree of myocardial fibrosis, and improvement in the heart's energy status [37]. This study will be completed on July 31, 2023.

All the precedent data point to the enormous potential that SGLT2-I has outside of its current use in diabetic control and point to positive results in several cardiovascular health measures in both diabetics and non-diabetics. As a result, this miraculous medication should be further investigated for its undiscovered advantages.

Limitations

We limited our search to just two databases that contained English-language studies published during the last five years; as a result, good publications from earlier years and in other languages would have gone undiscovered. Grey literature and other databases were not incorporated. As far as we are aware, there are no larger clinical trials with a purely non-diabetic population only. Furthermore, non-diabetic populations had a smaller sample size in recent studies. Most of the studies we discussed were post hoc analyses or prespecified subgroup analyses on previously completed bigger RCTs with a significant proportion of diabetes participants. Finally, we didn't discuss much of the side effects of SGLT2-I.

## Conclusions

In addition to its ability to lower blood glucose levels, SGLT2-I has been shown in various studies to improve patients' cardiovascular health. Therefore, this systematic review aimed to learn more about its application in non-diabetic cardiac patients, whether they had acute or chronic HFs. Even though no significant clinical trial has been carried out solely on the non-diabetic population, recent studies have demonstrated cardiovascular benefits in the cohorts of both diabetic and non-diabetic populations and have demonstrated a significant reduction in HF hospitalization and mortality from cardiovascular causes. The above-stated efficacy in both populations is identical, which is surprising. The outcomes of present and upcoming RCTs in patients with MI, atrial fibrillation, and hypertrophic cardiomyopathy will likely expand the clinical indications for SGLT2-I in a variety of patient categories, enabling us to explore their potential beyond anti-glycemic effects. Future research will, however, require more trials with bigger non-diabetic sample size.
